# Protocol for a multicentre randomized clinical trial comparing oncological outcomes of D2 *versus* D3 lymph node dissection in colonic cancer (COLD trial)

**DOI:** 10.1002/bjs5.50142

**Published:** 2019-03-14

**Authors:** A. Karachun, A. Petrov, L. Panaiotti, Y. Voschinin, T. Ovchinnikova

**Affiliations:** ^1^ Surgical Department of Abdominal Oncology National Medical Research Centre of Oncology named after N. N. Petrov St Petersburg Russia; ^2^ Pathology Department National Medical Research Centre of Oncology named after N. N. Petrov St Petersburg Russia

## Abstract

**Background:**

The extent of lymph node dissection in colonic cancer surgery remains arguable, and evidence from RCTs regarding extended lymph node dissection outcomes is lacking. This study aimed to compare the long‐term results of D3 lymph node dissection with those of D2 dissection.

**Methods:**

This is a multicentre RCT. The aim is to enrol 768 patients with primary colonic cancer assigned randomly to D2 or D3 lymph node dissection. The trial is assessing the superiority of 5‐year overall survival as the primary endpoint in patients undergoing D3 lymph node dissection *versus* D2 dissection. Secondary endpoints include disease‐free survival, short‐term outcomes (30‐day morbidity and mortality), quality of complete mesocolic excision and lymph node dissection, pattern of lymph node metastasis and quality of life in patients following D2 and D3 lymph node dissection. Experience of 20 D3 and 20 D2 lymph node dissections is required for surgeons to participate in the trial. For surgical accreditation four non‐edited videos of procedures will be assessed. Patients will be followed up for 5 years after last patient enrolment. Intention‐to‐treat analysis will be performed.

**Discussion:**

The results of this study will demonstrate whether extended lymph node dissection is superior to standard dissection in terms of oncological outcomes, and will also assess the impact of more extensive surgery on short‐term outcomes and quality of life.

## 1 Trial registration

**Table:** 

Primary registry and trial identifying number	Clinicaltrials.gov NCT03009227
Date of registration in primary registry	4.01.2017
Source of material support	National Medical Research Centre of Oncology named after N.N. Petrov
Primary sponsor	National Medical Research Centre of Oncology named after N.N. Petrov
Contact for public queries	Aleksei Karachun dr.a.karachun@gmail.com
Contact for scientific queries	Aleksei Karachun dr.a.karachun@gmail.com
Title	Multicentre randomized controlled study of oncological outcomes of D3 Lymph Node Dissection in Colon Cancer (COLD)
Countries of recruitment	Russia
Problem studied	Surgical treatment of colon cancer
Interventions	Active control: colon resection with D2 lymph node dissection Active comparator: colon resection with D3 lymph node dissection
Key eligibility criteria	Adult male and female above 18 years old signed informed consent with diagnosed primary colonic cancer. Inclusion criteria: pathologically confirmed adenocarcinoma of colon, TNM – T1‐4aN0‐2 M0‐1 (if metastases are resectable (see 8.2, 10.2)). Exclusion criteria: adjacent organs invasion, non‐resectable distant metastases, acute bowel obstruction, bleeding, tumour perforation (see 8·3).
Study type	Interventional randomized with parallel assignment, non‐blind, phase 3
Date of first enrolment	February 2017
Total sample size	768 patients
Recruitment status	Recruiting
Primary endpoint	Overall survival (5 years after last patient enrolled)
Secondary endpoints	Disease free survival (5 years after last patient enrolled), short‐term outcomes (30 days after operation), lymph node metastases pattern, quality of CME, lymph node yield, quality of life after D2 and D3 lymph node dissections

## 2 Sources of financial and non‐financial support

Surgical operations, diagnosis and treatment of patients and its financial support are included in the national healthcare system and are free for patients. Protocol development, data collection and analysis is financed under the routine funding of scientific work in National Medical Research Centre of Oncology named after N.N. Petrov.

## 3 Roles and responsibilities

### 3.1 Authors' contributions

Aleksei Karachun – concept of the study, study design and critical review of protocol drafts.

Aleksei Petrov – concept of the study, study design and development of protocol.

Lidiia Panayotti – literature review, calculation of sample size and statistical expertise and conducting of data analysis.

Yegor Voshchinin – critical review of protocol drafts.

Tatyana Ovchinnikova – development of pathology report protocol.

All authors contributed to refinement of the study protocol and approved the final manuscript.

### 3.2 Study sponsor

The study is sponsored by National Medical Research Centre of Oncology named after N.N. Petrov, 68 Leningradskaya Street, pos. Pesochniy, Saint‐Petersburg, Russia, 197758, contact no 7‐812‐4399555, e‐mail: oncl1@rion.ru, contact – coordinating investigator Aleksei Karachun.

### 3.3 Sponsor and funder

Funding sources had no role in the design of this study and will not have any role during its conduct and analysis.

### 3.4 Overseeing the study responsibilities

**Coordinating investigator:**

Control of the conduct of COLD trial.

Development of protocol and protocol amendments.

Development of Case Report Forms (CRFs).

Arrangements of steering committee meetings.

Preparation and revision of publications.

Study conduct reports preparation.

**Steering committee (coordinating investigator, principal investigators from each centre):**

Maintaining of planned enrolment.

Safety reports review.

Approval of protocol amendments.

Control of quality of study conduct in each centre.

Decision of continuation or termination of the study in centres.

## 4 Protocol synopsis

**Table:** 

Title:	Multicentre randomized controlled trial of oncological outcomes of D3 lymph node dissection in colon cancer
Study design:	*Patients with primary resectable colon cancer included in the trial are randomized into two groups for D2 (group I) and D3 (group 2) dissection*
Aims and endpoints:	*Aim of the study*: To determine if long‐term outcomes of D3 lymph node dissection in colon cancer are superior to D2 lymph node dissection *Primary endpoint*: Overall survival (time frame: 5 years after last patient enrolment)
	*Secondary endpoints*: Disease‐free survival (time frame: 5 years after last patient enrolment)
	Short‐term outcomes (time frame: 30 days after operation) Lymph node metastasis pattern (number of lymph node with metastases related to number of lymph nodes studied) CME quality (ratio of good, satisfactory and unsatisfactory quality) Lymph node yield (number of lymph nodes removed) Quality of life of patients
**Patients:**	*Male and female patients over 18 years old, who signed informed consent, with diagnosed primary colon cancer T1‐4a N any M 0‐1 (in case of resectable metastases)*
**Number of patients**	*768 (384 + 384)*
**Study duration:**	*The study is conducted starting from January 15 2017 until target number of patient is enrolled and 5 years of follow‐up after enrolment of the last patient*
**Estimated time until target number of patients is reached:**	*3 years*
**Statistical analysis**	*With ά value of 0,05 and β – 0,8, 768 patients enrolment is planned to determine statistical superiority of overall survival after D3 lymph node dissection over D2 dissection with assumed 10% increase in survival with 3 years accrual period and 5 years of follow‐up*

## 5 Background

D3‐lymphadenectomy was first proposed in Japanese guidelines for treatment of colon and rectal cancer in 1977^1^. Since that time it has been a surgical standard of care in Japan, China, Korea and Taiwan. It includes complete removal of paracolic, intermediate and central lymph nodes. In right side colon cancer D3‐lymphadenectomy involves removal of all groups of lymph nodes located along the main feeding vessels, which are branches of the superior mesenteric artery (ileocolic, right or middle colic artery). If colon bearing the tumour receives blood supply from branches of inferior mesenteric artery, the removal of lymph nodes located along the trunk of the vessel (the section between the place of origin on aorta and the left colic artery) is essential^2^. This dissection can be carried out precisely along the major vessels and Japanese guidelines do not highlight the need for bowel mesentery excision between embryological layers^3^. Thus, the Japanese guidelines put great emphasis on the level of vessels ligation and lymphadenectomy, but the layer in which the mesentery mobilization should be done is not described in detail.

In Europe, more emphasis is put on the principles of embryology‐focused surgery and on determination of mesentery mobilization layer. The concept of complete mesocolic excision (CME) has aroused much interest as a technique, which can reduce the incidence of local recurrence and increase survival in patients with malignant tumours of the colon. The concept of CME is a sequel of the concept of total mesorectal excision (TME), a recognized and widely used technique in rectal cancer surgery. Implementation of TME into a wide surgical practice has reduced local recurrence rate and increased survival^4^. In TME the dissection is carried along the mesorectal fascia. In CME the layer of surgical dissection lies between the visceral and parietal fascia. So, both techniques are based on principles of embryology‐focused surgery and ensure the idea of en‐bloc resection.

CME concept proposed by Hohenberger et al in 2009 and demonstrated in Erlangen, can be considered as traditional Western approach^5^. According to the method of CME, a key aspect of the operation is sharp and precise division of embryological layers ‐ mobilization of the mesentery along Told's fascia, preserving its integrity and removing all the containing lymph nodes. The level of vascular ligation and groups of lymph nodes to be removed are not clearly defined. Usually CME is performed with CVL (central vascular ligation) ‐ high ligation of vessels. CVL is not an equivalent to D3: feeding vessel may be ligated either at the base, or at a distance of up to 1 cm from the point of origin^6^.

Thereby, the western and eastern concepts of colon resection are similar to a certain extent, but emphasize different aspects of the operation. Japanese approach ‐ D3 lymphadenectomy means high precise definition of lymph node groups to be removed, without mentioning the dissection layer; The European approach ‐ CME, on the other hand, describes in detail the dissection along embryological layers, but does not identify the level of vascular ligation clearly. The main difference is the amount of resected bowel. Comparing specimens from the clinic in Erlangen and from 2 Japanese hospitals revealed that in Japan less colon is resected as well as the number of lymph nodes removed is smaller. This is due to the fact that the Japanese hold the position of ‘economical’ removal of the organ, and the Europeans perform standard hemicolectomies and resection of the colon^7^.

There are various combinations of the above‐mentioned surgical techniques. CME can be combined with any level of vascular ligation, as well as each level of vascular ligation can be complemented by en‐bloc resection following the concept of CME^8^.

Proponents of CME with CVL believe that this method complies with all the principles of oncology, and its main advantage is in the removal of the maximum number of lymph nodes. This contributes to more accurate staging of the disease and the appointment of adequate adjuvant therapy^9^, ^10^. Removal of 28 lymph nodes and more is associated with better survival even in N0 patients^5^. Performing CME helps to remove virtually all of nodal metastases^11^. Fascial envelope damage, on the contrary, leads to deterioration in survival^12^, ^13^. Another advantage of CME is the possibility to standardize the surgical technique of colon cancer treatment and thus improve its quality^14^.

There is a contrary view on the extent of lymphadenectomy. Some authors believe that removal of all mesenteric lymph nodes is unnecessary, and the effect of lymphadenectomy is unproven^15^, ^16^. Some authors proclaim that positive results after CME with the CVL are nothing but a consequence of a good surgical technique^17^. However, expansion of dissection leads to more postoperative complications and functional disturbances than observed after standard resection^18^. For example, high ligation of ileocolic artery can lead to severe diarrhoea; para‐aortic dissection and high ligation of the inferior mesenteric artery may damage the hypogastric plexus and cause sexual dysfunction and urological problems^6^.

Several authors have published data on the significant reduction of local recurrence rate and increase in 5‐year survival after the CME with CVL^5^, ^19^, ^20^, ^21^, ^22^, ^23^, ^24^, ^25^, ^26^, ^27^. No increase in morbidity or mortality was found in these studies. Long‐term results after CME were demonstrated by Hohenberger et al in 2009. Reduction in local recurrence rates from 6·5% to 3·6% was shown. 5‐Year cancer‐related survival improved from 82·1 to 89·1%^5^. However, this data is the result of comparison of patients treated in 1978‐1984 with patients treated in 1995‐2002, which makes it impossible to exclude influence of other factors on the outcome.

Standardization of CME with CVL techniques allowed Bokey et al. to observe improvement in survival rate from 48 to 63% and the cancer‐specific survival from 66 to 76%^26^.

Storli et al. analysed CME outcomes in early colon cancer T1‐2 N0—the overall 3‐year survival after CME was 88·1% (79% in the comparison group), disease‐free survival was 82·1% (79% in the comparison group)^21^.

Kotake et al. were studying outcomes of D3 lymph node dissection in locally advanced colon cancer on the data from Japanese database. It was shown that extended lymph node dissection is associated with increased overall survival and median lymph node yield (21·8 versus 14·9)^28^.

Of interest are the results obtained by Bertelson et al. when comparing a group of patients who underwent CME to those who underwent conventional resection. They showed an increase in 4‐year disease‐free survival for the CME group (85·8 and 75·9%, respectively), but no statistically significant differences were found in overall survival. Increase in disease‐free survival was observed only in patients with stage I and II disease, but not III. They also demonstrated reduction of local recurrence rate after CME (11·3% vs. 16·2% for the comparison group). Multivariate regression analysis showed that CME is an independent predictor of disease‐free survival increase for patients with I ‐ III stages disease.

West et al. used the criteria from the MRC CLASSIC trial to study the plane of mesentery resection for colon cancer. Similarly to the principles of TME evaluation the authors identified three possible planes of dissection: mesocolic, intramesocolic and muscularis propria layer. It was found that only 32% of the dissections were performed in mesocolic layer. They also showed 15% survival advantage for mesocolic plane of resection compared to a muscularis propria plane^12^.

There are at least three ongoing randomized trials studying extent of surgery for colon cancer. LCME trial is comparing lymph node yield after laparoscopic colonic resections with CME vs. D3 lymph node dissection. Mean lymph node count is 20·3 ± 5·8 in CME vs. 19·2 ± 6·7 in D3^29^. Long‐term results are awaited. RELARC trial is researching the hypothesis of superiority of disease free survival after laparoscopic D2 lymph node dissection compared to CME for right colon cancer^30^. SLRC trial is assessing disease free survival after laparoscopic CME vs. D3 lymph node dissection for right colon cancer^31^.

In the studies mentioned there is a certain lack of unity in terminology and concept as in different studies CME is opposed to D2 and D3. In COLD trial the CME is the concept of sharp dissection and is a standard in both groups, while randomization determines the level of vascular ligation. Right and left colon cancers are included, and mini‐invasive or open approach can be used.

Thus, the principles of embryology‐focused surgery and research data underline the need to perform the mesentery resection in the mesocolic layer routinely to improve the results of surgical treatment of patients with tumours of the colon. At the same time the advantages of extended lymphadenectomy (removal of groups of lymph nodes related to the level of D3) are debated. Short‐term and long‐term results of the D3 dissection for cancer colon cancer require further research and comparison to the results of D2 lymphadenectomy.

## 6 Aims of the study

### 6.1 Primary aim of the study

To determine if long‐term outcomes of D3 lymph node dissection in colon cancer are superior to D2 lymph node dissection.

### 6.2 Secondary aims of the study

To determine if there are any differences in disease‐free survival, short‐term outcomes after D2 and D3 lymph node dissections, assess quality of CME, lymph node yield and patients' quality of life.

## 7 Study design

### 7.1 Design

This is a randomized controlled phase III study, comparing long‐term outcomes of D2 and D3 lymph node dissections in colon cancer. Eligible patients signed written informed consent will be randomized for D2 or D3 lymph node dissection in 1 : 1 ratio.

After in‐hospital treatment postoperative recovery parameters will be evaluated, after that there will be a 5‐year follow‐up period. 768 patients will be recruited in registered centres in Saint‐Petersburg, Moscow and other centres (including other countries if any), registered for the study after patient enrolment had been commenced (up‐to‐date information about centres location can be found on https://clinicaltrials.gov/ct2/show/NCT03009227#contactlocation). Centres are activated after registration procedure (see 9·1). Patients are enrolled via screening procedure and assessing eligibility criteria. To obtain enrolment of planned number of patients additional centres will be initiated and an intensive contact with investigators in each centre will be stimulating desired enrolment.

### 7.2 Randomization

Randomization will be performed via main centre (National Medical Research Centre of Oncology named after N.N. Petrov). Eligible patients will be randomized for D2 or D3 lymph node dissection in 1 : 1 ratio for D2 or D3 dissection according to computer generated randomization list created in beforehand^32^.

Information regarding restrictions of randomization to provide groups' homogeneity is located in the document “Mechanism of randomization in COLD trial”, kept in the main centre of the trial concealed from investigators enrolling patients.

Randomization will be performed via sending of randomization list (filled in after patient's enrolment) by electronic message to main centre, where patient will be assigned to a certain line in the randomization list. In reply message investigator receives randomization group and code of the line in the list.

There is no blinding of investigators or patients in current study.

### 7.3 Endpoints

Primary endpoint:

- Overall survival (time frame: 5 years after last patient enrolment)

Secondary endpoints:

- Disease‐free survival (local recurrence or distant metastases during study period)
- Short‐term outcomes (time frame: 30 days after operation)
- Lymph node metastasis pattern (number of lymph node with metastases related to number of lymph nodes studied in each lymph node group)
- CME quality (ratio of good, satisfactory and unsatisfactory quality)
- Lymph node yield (number of lymph nodes removed)
- Quality of life of patients

## 8 Study population

### 8.1 Study population

The study is conducted among male and female patients older than 18 years old, who signed the informed consent form with diagnosed primary colon cancer T1‐4a N0‐2 M0‐1 (in case of resectable metastases). All patients should meet all inclusion criteria and have no exclusion criteria.

### 8.2 Inclusion criteria

1. Histologically confirmed adenocarcinoma of the colon (caecum, ascending, hepatic flexure, transverse colon, splenic flexure, descending colon, sigmoid).
2. TNM stage according to VII version T1‐4aN0‐2 M0‐1 (M1 in case of resectable metastases (see 10·2) confirmed by preoperative staging (colonoscopy, CT chest and abdomen).
3. Clinical indications for colonic resection.
4. ECOG status – 0‐2.
5. Age above 18 years old.
6. Signed informed consent.

### 8.3 Exclusion criteria

1. Medical or psychiatric reasons interfering with patient's decision to participate in the study.
2. Pregnancy or breastfeeding.
3. Medical conditions contraindicating surgical procedure.
4. Synchronous or metachronous malignancies.
5. Invasion to adjacent organs.
6. Non‐resectable metastases.
7. Acute bowel obstruction, bleeding or perforation.
8. Indications for isolated transverse colon resection.
9. Neoadjuvant chemotherapy.

## 9 Quality control

### 9.1 Centre registration

Centre registration procedure is initiated after centre principal investigator's application. Not less than 50 colonic resections for cancer are to be performed annually in the centre. Conditions to perform staging, treatment, morphological assessment and follow‐up according to current protocol are required. Local ethical committee approval is needed.

### 9.2 Surgeon registration

To participate in the study a surgeon should have an experience of not less than 20 D3 and 20 D2 lymph node dissection procedures for colon cancer. Surgeons are separately registered for open and laparoscopic procedures. Unedited videos of surgical procedures are required for right hemicolectomy and left (or sigmoid) hemicolectomy – two for each with D2 and D3 lymph node dissections (video assessment can be omitted if coordinating investigator of the study observed mentioned amount of procedures in person). After revision of the application additional videos may be required for assessment by coordinating investigator or a designated expert. Key points for assessment of surgical quality include, but are not limited by the principles noted in *Appendix* S1 (supporting information). Registered for the study surgeon should be scrubbed and take part in the procedure from the beginning of mobilization to the end of anastomosis formation. Participation of the registered surgeon as an assistant is possible. Presence of the registered surgeon in the operating room not being in the scrubbed team is not allowed.

### 9.3 Quality of surgical procedures control

To provide routine quality control in every centre photographs of each procedure are required to assess the lymph node dissection level and vessels ligation – for instance, photos containing vessels with a clip prior to transection.

### 9.4 Morphological assessment

Groups of lymph nodes are to be marked on a non‐fixed specimen by a member of the surgical team taking part in the procedure determined by the registered surgeon. Groups are marked according to performed dissection level using Japanese classification (*Fig*. 1, *Appendix* S1, supporting information). Proximal part of 252 group between left colic and first sigmoid artery is marked as 252A and the part between the first sigmoid and 251 group is marked as 252B.

Specimen assessment is performed using principles, described by N. West et al.^12^ Full protocol of pathology assessment is described in *Appendix* S2 (supporting information).

## 10 Patient treatment

### 10.1 Preoperative care

Preoperative investigations and co‐morbidities treatment is performed according to centre's guidelines and protocols. Preoperative staging is to include, but not limited to IV contrast CT chest and abdomen (or abdominal MRI if CT is contraindicated), colonoscopy, histological confirmation of cancer, cancer embryonic antigen level.

If a female patient is not in menopause or it has been less, than 2 years after menopause, a pregnancy test is performed. Results of investigations and conclusion if a patient meets eligibility criteria are documents in case report file.

If a treatment of co‐morbidities is required prior to surgery it is performed before the patient is included.

### 10.2 Patient enrolment

Patients are enrolled by investigating physician. Study is discussed with the patient and time and opportunity is given for the patient to familiarize with the informed consent. Patient's consent is confirmed with the signature. After informed consent is signed patient is considered as included in the trial.

For patients with resectable metastases before enrolment treatment plan of metastases removal should be formulated and documented (simultaneous operation or staged procedures). If metastases cannot be resected, such patients are not included in the trial.

Patients meeting all inclusion criteria and having no exclusion criteria are randomized for D2 or D3 lymph node dissection (see 7·2).

### 10.3 Surgery

Surgical operation is performed under general anaesthesia with or without spinal or epidural. During screening a registered surgeon determines necessary extent of colonic resection and planned approach in accordance with the type of surgery for which the surgeon was registered. Vessels, which are to be ligated according to tumour location, are documented. These vessels are to be dissected in D2 or D3 area depending on randomization group. Randomization group does not interfere with colonic resection extent, but only determines the area of ligation of vessels mentioned before randomization. Steps of surgery and extent of lymph node dissection are described in *Appendix* S1 (supporting information).

Date of surgery, time of start and end of surgery, blood loss, blood transfusions and approach are registered. In case of conversion, reason of conversion is noted: “tactical” conversion is performed after laparoscopic revision in case of new data, complicating laparoscopic procedure (adhesions, tumour size etc.), “reactive” conversion is understood as a change in approach because of intraoperative events (bleeding, perforation etc.). Intraoperative findings, main steps and type of surgery are registered in operative notes and in electronic CRF (eCRF). In case of multiorgan resections the extent and details of other organ resections are documented.

Surgeon's impression on performed extent of lymph node dissection, quality of CME and quality of surgery in general is registered noting if lymph node dissection was compliant with the randomization group. Photos of ligated vessels prior to transection are attached. If performed lymph node dissection differed from the randomization group, reason is explained.

Surgical wound closure details are documented.

### 10.4 Postoperative care

Postoperative treatment is given according to guidelines accepted in a centre. Main parameters of postoperative period are registered in patient's eCRF: admission and discharge dates (if the discharge is postponed for social reasons ‐ date, when patient was medically fit for discharge is documented), day of first bowel peristalsis, passing wind, opening bowels, start of fluids and soft diet, patients movement activity, blood transfusions. Complications are registered according to Clavien–Dindo classification. Treatment for complications is given according to local centre guidelines. Data on beginning, duration and treatment for complications is documented in patients' eCRF.

### 10.5 Chemotherapy

Chemotherapy is given according to local guidelines and indications in the centre. Neoadjuvant chemotherapy is an exclusion criterion. After pathology report on removed specimen a decision on necessity of adjuvant treatment is made. As chemotherapy is given as a part of common treatment and is not a matter of research indications and contraindications (among them avoidance of pregnancy during treatment) are determined by a doctor prescribing chemotherapy according to current guidelines and recommendations. Data on all types of treatment is documented in eCRF.

### 10.6 Follow‐up

Postoperative follow‐up includes investigations (CT chest and abdomen, colonoscopy) in 6 and 12 month, afterwards annually during the follow‐up period and examinations (or if in‐person contact is not possible it can be substituted by a phone call) in 30 days after surgery and afterwards every 3 month during follow‐up period. During follow‐up period complications, adjuvant treatment and recurrence are registered. Recurrence is registered when local recurrence or distant metastases are found during investigation (Colonoscopy, CT, MRI); serology recurrence is registered if CEA is 3 times higher than baseline. Quality of life will be assessed with validated questionnaires EORTC QLQ‐C30 and colorectal module CR 29 according to *Appendix* S3 (supporting information).

Patients rejecting surgery after randomization or patients receiving different to randomization group treatment will be analysed in primary randomization groups. For patients rejecting treatment and follow‐up continuation of data collection without treatment is offered.

## 11 Efficacy and safety

Efficacy and safety of surgical procedures will be assessed basing on pathology report, postoperative period data ‐ morbidity and mortality. Safety analysis is performed every 100 enrolled patients. Deviation of morbidity and mortality or frequency of adverse events for more than 30% in extended dissections group will be discussed at steering committee meeting and decision whether to continue or stop the trial will be made.

Surgery risks, preferable approach and conversion decision is surgeon's responsibility.

Adverse event is understood as any unwanted medical deviation in a patient receiving any kind of treatment not necessarily associated with the treatment. In current study adverse event is any unfavourable or unwanted deviation including laboratory tests or condition not necessarily associated with the surgery. Adverse event ‐ any change from preoperative state of a patient, including exacerbation of co‐morbidities.

Serious adverse event ‐ any adverse event resulting in: death, life‐threatening condition, requiring hospital admission or prolongation of in‐hospital treatment, permanent disability or incapacity.

Hospital admissions or deaths due to progression of the disease are not considered to be serious adverse events. Adverse events are described and rendered in eCRF. Relation of adverse events to surgical procedure is determined by investigator. Adverse events classification is performed in accordance with CTCAE ver. 4·0 terminology.

Any treatment or observation in case of adverse event is prescribed by attending doctor, who is responsible for it.

## 12 Follow‐up

Schedule of necessary investigations and follow‐up visits is in *Appendix* S3 (supporting information).

Visit day date deviation: 30 day visit +/‐ 2 days, other visits ‐ +/‐ week. Data on completed visits is transferred to eCRF in 5 working days. Quality of life is assessed via questionnaires filled in before surgery, on day 30, 3‐month visit, 1, 3 and 5‐year visits.

To minimize number of patients lost for follow‐up patients are able to provide results of their routine follow‐up investigations and get a consult of expert centre, which will facilitate patients' adherence to follow‐up.

## 13 Data quality

To provide high quality of data regular on‐site monitoring visits will be arranged.

Data is entered to electronic CRF and after the visit is marked as complete data is verified during monitoring visit.

Monitoring visits are scheduled according to number of visits ready for verification. Date of the visit is agreed with centres in advance.

Before initiation of the trial an interactive training is conducted and a test electronic database is created for familiarization with the system and test data entry.

During monitoring visits primary medical documentation and eCRF data are matched. After verification of the data it can be exported for the analysis.

Monitors also ensure the consent, ethical committee approval and other relevant documents are present.

Data of all patients is monitored. First monitoring visits are verifying 100% of data, after analysis of monitoring visits «risk assessment monitoring» may be introduced.

In case of any problems with trial conduct in a centre monitors facilitate resolving of such problems.

**Table:** Superiority by a Margin Test for the Difference of Two Exponential Hazard Rates Analysis

Numeric Result with Ha: h2 < h1 ‐ Δ and Uniform Accrual											
	Total Sample Size	Control Sample Size	Trtmnt Sample Size	Control Hazard Rate	Trt Hazard Rate	Clin'l Super'ty Bndry	Control Loss Hazard Rate	Trtmnt Loss Hazard Rate	Accrual Time	Follow Up Time	
Power	N	N1	N2	h1	h2	B	ω1	ω2	R	T‐R	Alpha
0,8004	768	384	384	0,139	0,102	0,139	0,165	0,165	3,0	5,0	0,050
Second Section of Numeric Report											
	Total Events	Control Events	Trtmnt Events	Prop'n Control N1/N	Hazard Rate Diff h2‐h1	Hazard Ratio h2/h1	Clin'l Super'ty Margin	Clin'l Super'ty Ratio	Var'nce of h1 hat	Var'nce of h2 hat	
Beta	E	E1	E2	P1	D	HR	Δ	r	σ^2^(h1)	σ^2^(h2)	
0,1996	270	150	120	0,500	‐0,036	0,737	0,000	1,000	0,049	0,033	

## 14 Collection, storage and sharing of data

Clinical data (demographics, diagnosis, investigations, surgery data, pathology, lab results etc.) is entered to eCRF basing on primary medical documentation (patient file, outpatient file, investigation reports, laboratory reports, operation notes etc.). For assessment of quality of life validated questionnaires EORTC QLQ‐C30 and colorectal module CR 29. Questionnaires are filled in during visits (face to face or on the phone).

Data in eCRF is stored under a pseudonym with password‐protected access on the server of main study centre. Data is stored for at least 15 years after the end of the study. Investigators have access to their centre's patients data, coordinating investigator and monitors have access to all patients' data.

Full access to final database will be available for coordinating investigator, access to centre's database is provided for relevant principal investigators on demand.

After verification data is exported for statistical analysis using SPSS software.

Publications in relevant journals of protocol, first safety report, short‐term results after enrolment is finished and final publication, when primary end‐point is reached, are planned. This data will also be shared among specialists via presentations on conferences.

## 15 Sample size calculation and statistical analysis

To determine the sample size current data from national cancer registry was used^33^, according to which 5 year survival of colon cancer patients is 50%. Survey among surgeons revealed anticipated improvement of survival due to D3 lymph node dissection of 10%. For sample size calculation MedCalc software was used (function of sample size calculation for survival analysis (log rank test) using D. Machin method^34^), according to current survival data, sample size was determined as 762 patients. Using sample size calculation for superiority study via PASS software with C. Chow et al and J. Lachin et al method^35^, ^36^, with α – 0,05 and β – 80%, with 15% of patients lost for follow‐up in case of 3 year accrual period and 5 year follow‐up sample size was determined as 768 patients. As sample sizes obtained by different methods were similar the greater number was used to determine the sample size as 768 patients.

“Intent‐to‐treat” analysis will be performed, thus once randomized patients will be analysed in their randomization group, irrespective of actually received treatment.

For statistical analysis of primary endpoint (overall survival during the study period) Kaplan–Meier method will be implied as the probability of patients survival depends only on time after surgery, as there is no anticipated change of survival during the trial conduct. To compare survival curves long‐rank test will be used.

For secondary endpoints type of analysis will depend on the nature of data. For disease‐free survival (survival without local or distant recurrence during the study period) Kaplan–Meier curves will be used with the long‐rank test. For analysis of short‐term outcomes (morbidity and mortality), lymph node yield parametric and non‐parametric criteria will be used to compare mean values and contingency tables.

For categorical data, to assess the significance of association of two kinds of classification (contingency tables) exact Fisher's test will be implied by default as it can be used when sample distribution is only approximately equal to theoretical distribution and therefore this test is universal. For the final analysis as the sample reaches calculated size Pearson Chi square test will be used.

Prior to comparing of group mean values in case of quantitative data, a non‐parametrical criterion of Shapiro Wilks will be used to test the normality of distribution. In case of normal distribution Student's criterion for independent samples will be used (dependant samples for quality of life assessment). For data not distributed normally non‐parametric Mann–Whitney U criterion will be implied.

Lymph node metastases pattern will be assessed as calculation of frequency of affected nodes related to number of studied nodes in each group. Correlation analysis of association of size, T‐stage, localization of the tumour with affected groups of lymph nodes, frequency of metastases, tumour budding grade, lymphovascular and perineural tumour invasion. Spearman rank correlation coefficient will be used as the data may be distributed not normally and numeric data may be included. To assess correlation of quantitative data distributed normally Pearson correlation coefficient will be implied.

For the primary endpoint and disease free survival missing data will be handled according to the survival analysis nature – censoring of the patient with the date of last contact.

Missing data for the secondary endpoints will be dealt with by using multiple imputations with Markov chain Monte Carlo method (monotone method if the data shows a monotone pattern of missing values; otherwise, fully conditional specification).

Conclusion of superiority of D3 lymph node dissection will be made in case of statistical superiority of survival during the study of D3 group over D2.

## 16 Control of study conduct

Study conduction is controlled by monitoring timing and quality of eCRF completion by representatives of main centre in National Medical Research Centre of Oncology named after N.N. Petrov, according to part 11 of the protocol to ensure patients safety short‐term outcomes will be monitored.

In case of protocol deviation during the study main centre may suspend any centre's or surgeon's registration in the study.

The protocol of the study and all amendments, as well as informed consent are to be approved by Ethical committee of National Medical Research Centre of Oncology named after N.N. Petrov. Prior to start of the study in a certain centre, approval of local ethical committee is to be obtained.

After amendments are approved, new version of protocol with changes description is spread among principal investigators.

## Supporting information


**Appendix S1** Principles of surgical procedures
**Appendix S2** Pathological evaluation of specimen
**Fig. 1.** Positioning of the specimen, lymphovascular pedicle and mesentery location
**Fig. 2.** Measurement of distance from artery ligation level to colon wall or tumour
**Fig. 3** Measurement of mesentery area and CME quality assessment
**Fig. 4** Transection of the bowel from both ends up to tumour level avoiding transection the latter.
**Fig. 5** Introduction of the swab to facilitate formalin delivery to the tumour
**Fig. 6** Swab is located in unopened part of the lumen
**Fig. 7** Circumferential resection margin staining
**Appendix S3** Visits schedule
