# Supplementary material for: Protocol for a multicentre randomized clinical trial comparing oncological outcomes of D2 versus D3 lymph node dissection in colonic cancer (COLD trial)
Source: BJS Open. 2019 Mar 14;3(3):288–98. doi: 10.1002/bjs5.50142 (PMC6551411; doi:10.1002/bjs5.50142)
Supplement: Supplementary file 1 — Appendix S1 Principles of surgical procedures Appendix S2 Pathological evaluation of specimen Fig. 1. Positioning of the specimen, lymphovascular pedicle and mesentery location Fig. 2. Measurement of distance from artery ligation level to colon wall or tumour Fig. 3 Measurement of mesentery area and CME quality assessment Fig. 4 Transection of the bowel from both ends up to tumour level avoiding transection the latter. Fig. 5 Introduction of the swab to facilitate formalin delivery to the tumour Fig. 6 Swab is located in unopened part of the lumen Fig. 7 Circumferential resection margin staining Appendix S3 Visits schedule [file BJS5-3-288-s001.docx]

**BJS5_50142**

**Protocol for a multicentre randomized clinical trial comparing oncological outcomes of D2 *versus* D3 lymph node dissection in colonic cancer (COLD trial)**

**A. Karachun, A. Petrov, L. Panaiotti, Y. Voschinin and T. Ovchinnikova**

# Appendix S1 Principles of surgical procedures

Main principles of surgery include, but are not limited to:

• precise sharp dissection along embryological layers (principles of complete mesocolic excision)

• safe cancer clearance - vessel ligation not distal to D2, clearance from the tumour not less than 5 cm of the colon

 • if metastatic proximal lymph nodes are revealed and they are feasible for removal - dissection of metastatic lymph nodes is recommended for any randomization group

 • hand sewn or stapled tension free anastomosis, safe mobilization of hepatic and splenic flexures

D2 lymph node dissection definition:

D2 lymph node dissection includes removal of mesocolic level of lymph nodes:

• in right hemicolectomy ileocolic artery and right colic artery are ligated - 202 and 212 groups of lymph nodes are removed (Classification of Japanese Colorectal Society, *Fig. 1*)

• in extended right hemicolectomy ileocolic, right colic and middle colic arteries are ligated - 202, 212 and 222 groups are removed

• in left hemicolectomy inferior mesenteric artery is ligated - 232, 242, 252 groups are removed (left colic artery may be ligated just at the origin instead, in that case 242 and 252 groups are not removed)

• in extended left hemicolectomy inferior mesenteric and middle colic arteries are ligated - 232, 242, 252 and 222 groups are removed (left colic artery may be ligated just at the origin instead, in that case 242 and 252 groups are not removed)

• in sigmoid resection inferior mesenteric artery is ligated - 242 and 252 lymph nodes are removed

D3 lymph node dissection definition:

For D3 lymph node dissection central (D3) groups of lymph nodes are to be removed

• in right hemicolectomy ileocolic and right colic arteries are ligated - 202, 212, 203 and 213 groups are removed

• in extended right hemicolectomy ileocolic, right colic and middle colic arteries are ligated - 202, 212, 222, 203, 213 and 223 groups are removed

• in left hemicolectomy inferior mesenteric artery is ligated - 232, 242, 252 and 253 lymph nodes are removed. Inferior mesenteric artery can be skeletonized with removal of 253 group en bloc with the specimen and left colic artery is ligated at the origin then (252 and 242 groups are not removed in that case)

• in extended left hemicolectomy inferior mesenteric artery and middle colic arteries are ligated - 232, 242, 252, 222, 253 and 223 groups are removed. Inferior mesenteric artery can be skeletonized with removal of 253 group en bloc with the specimen and left colic artery is ligated at the origin then (252 and 242 groups are not removed in that case)

• in sigmoid resection inferior mesenteric artery is ligated - 242, 252 and 253 lymph nodes are removed.

During both types of lymph node dissections paracolic lymph nodes of resected colon segment are removed.

Therefore, extended hemicolectomy is defined as a procedure, when according to tumour location middle colic arteries are to be ligated. Depending on level of ligation of the vessels extended hemicolectomy can be performed with D2 or D3 dissection.


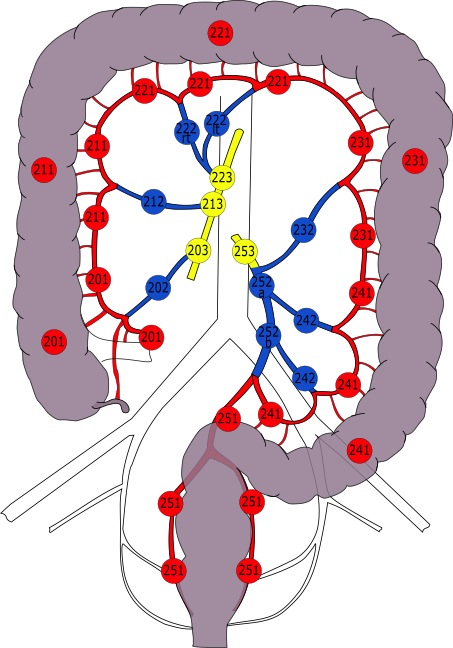


***Fig. 1 Classification of colon lymph nodes (Japanese Colorectal Society classification)***

Following landmarks are used to assess quality of surgery:

***Left colon and sigmoid:***

**D2** – inferior mesenteric artery is ligated just proximal to left colic artery origin. Structures deeper than Told's fascia (aortic wall and para-aortic nerve plexus) are not revealed.

**D3** – inferior mesenteric artery is dissected up to aorta and aortic wall is exposed, 253 group is moved to the specimen, dissection is performed beyond embryologic layer at this point to expose aortic wall.

***Right colon:***

**D2** – ileocolic artery is ligated on the level of mesentery root centrally to lower border of duodenum; superior mesenteric vein wall is not exposed. If right colic and/or middle colic artery are to be ligated, they are transected on the edge of mobilized mesentery without exposure of superior mesenteric vein

**D3** – anterior surface of superior mesenteric vein exposure is obligatory. Segment of artery to be ligated is exposed in the area between superior mesenteric artery and vein. Arteries are transected at the place of origin with visualization of superior mesenteric artery wall and removal of 203, 213 and 223 (for extended right hemicolectomy) groups of lymph nodes en bloc with specimen.

# Appendix S2 Pathological evaluation of specimen

1. Preparation of specimen for sectioning

Surgical specimen is delivered to pathology department with clear form, containing patient's name, age, diagnosis, type of surgery, extent of lymph node dissection performed, groups of lymph nodes are to be marked by surgical team (tags with numbers of groups of lymph nodes are to be attached to relevant parts of mesentery). Pathologist positions the specimen (*Fig 1*), assesses the quality of CME and performs following measurements: length of resected bowel (for right side small and large bowel separately), area of resected mesentery (if mesentery has trapezoid shape – trapezoid square formula can be used), distance from artery ligation to colon wall and distance from artery ligation to tumour (*Fig. 2*). If the edge of the tumour cannot be located colon may be locally transected to reveal tumour edge.


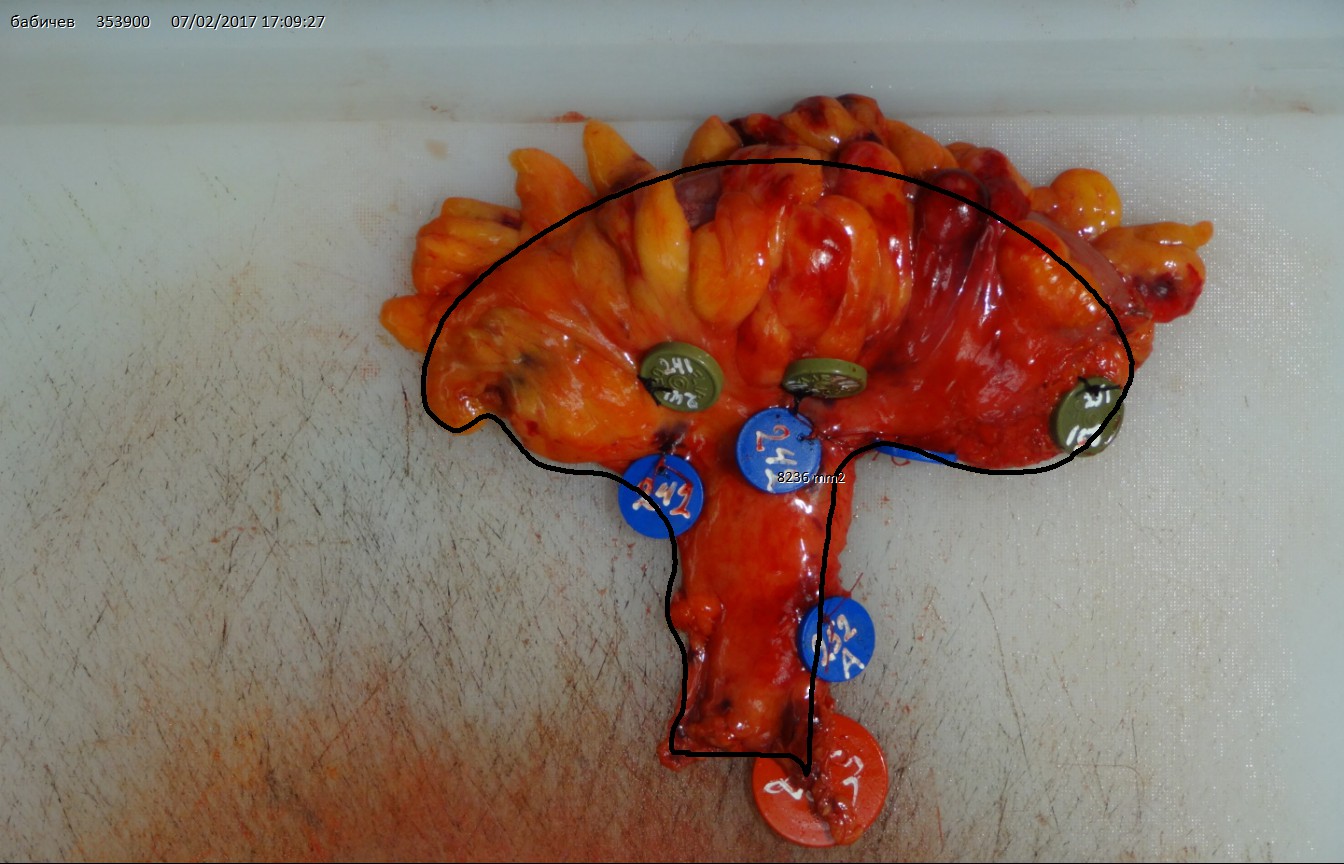


Fig. 1. Positioning of the specimen, lymphovascular pedicle and mesentery location


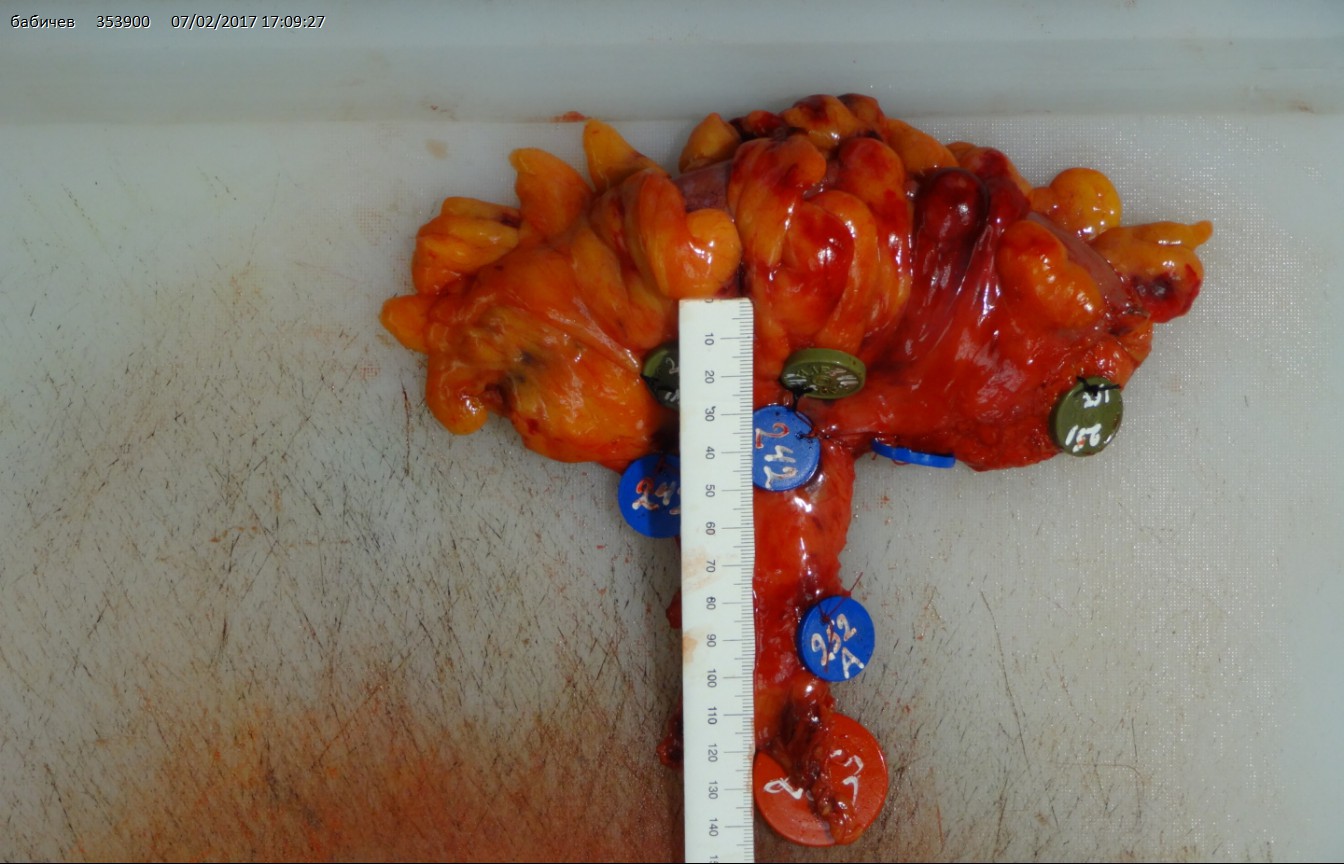


Fig. 2. Measurement of distance from artery ligation level to colon wall or tumour

Measurement of area of mesentery is performed as the specimen is photographed after mesentery is lying flat on even surface (*Fig. 3*). CME quality is assessed according to TME specimen concept: good quality – smooth fascia, defects less than 5 mm depth, satisfactory – defects deeper than 5 mm not reaching muscularis propria, unsatisfactory quality – deep defects reaching muscularis propria.


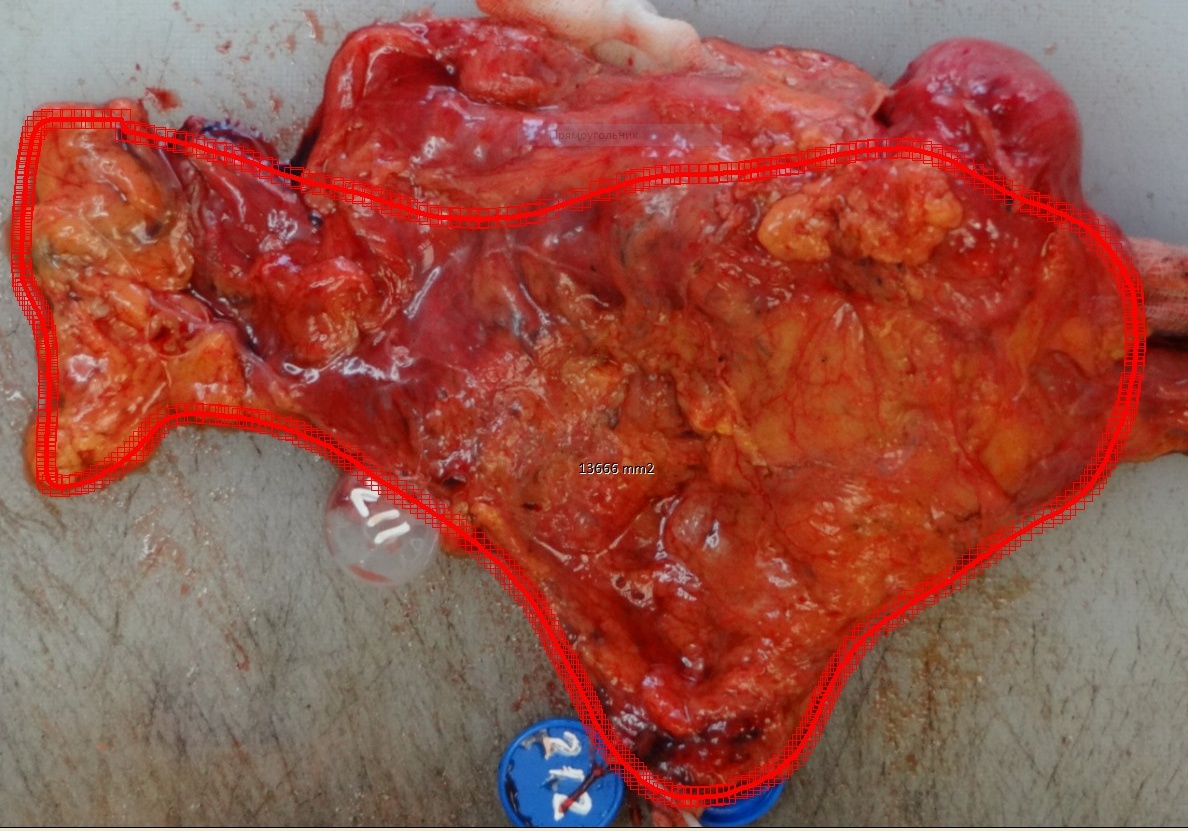


Fig. 3 Measurement of mesentery area and CME quality assessment

Then the bowel is transected on the free from mesentery edge from both ends up to tumour to open the lumen avoiding transecting the tumour. Proximal and distal margins are measured (*Fig. 4*).

After measurements a swab is inserted into the lumen where the tumour is located to facilitate formalin delivery to tumour for proper fixation of the specimen.


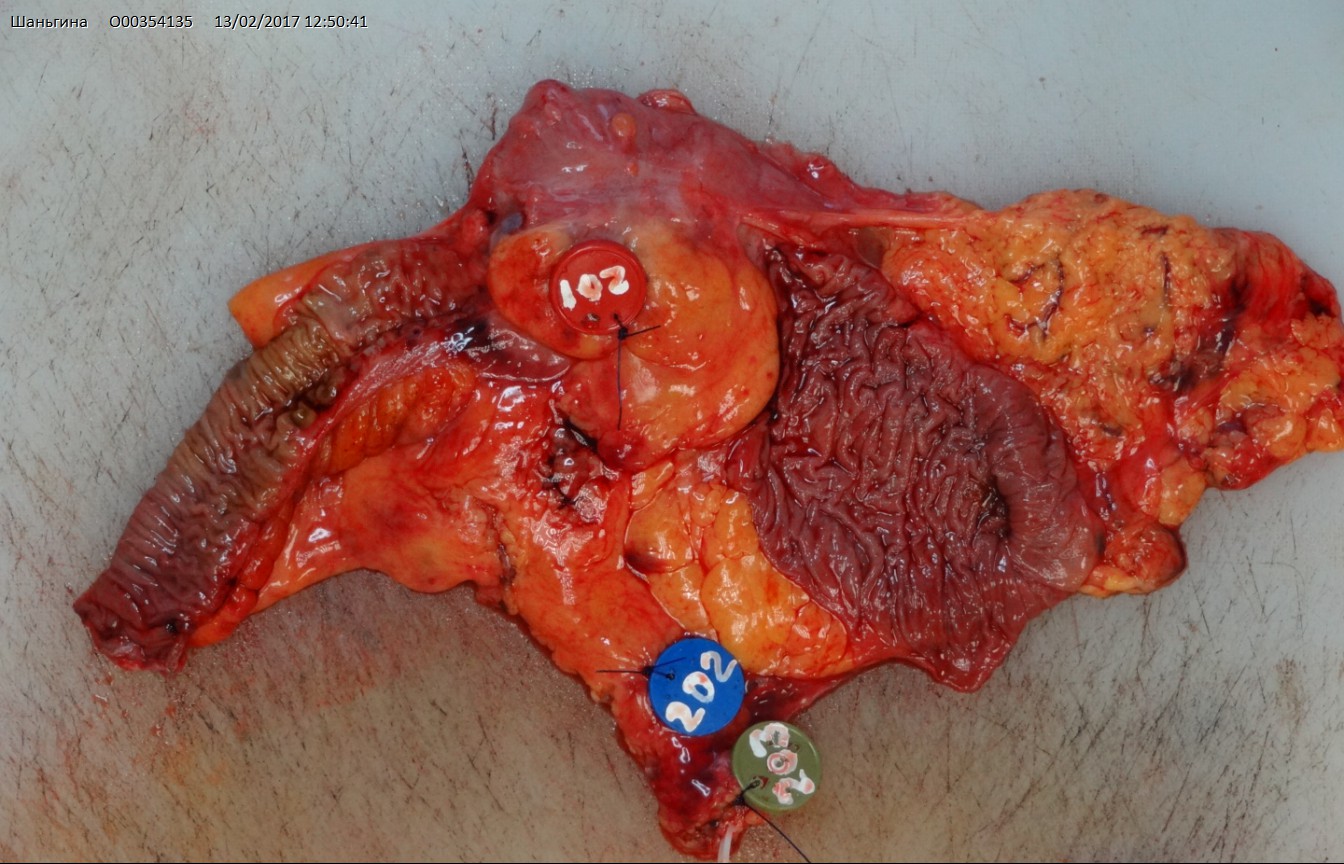


Fig. 4 Transection of the bowel from both ends up to tumour level avoiding transection the latter.

Forceps are introduced through the lumen; a swab is grasped and pulled through the channel in the lumen (*Figs 5* and *6*).


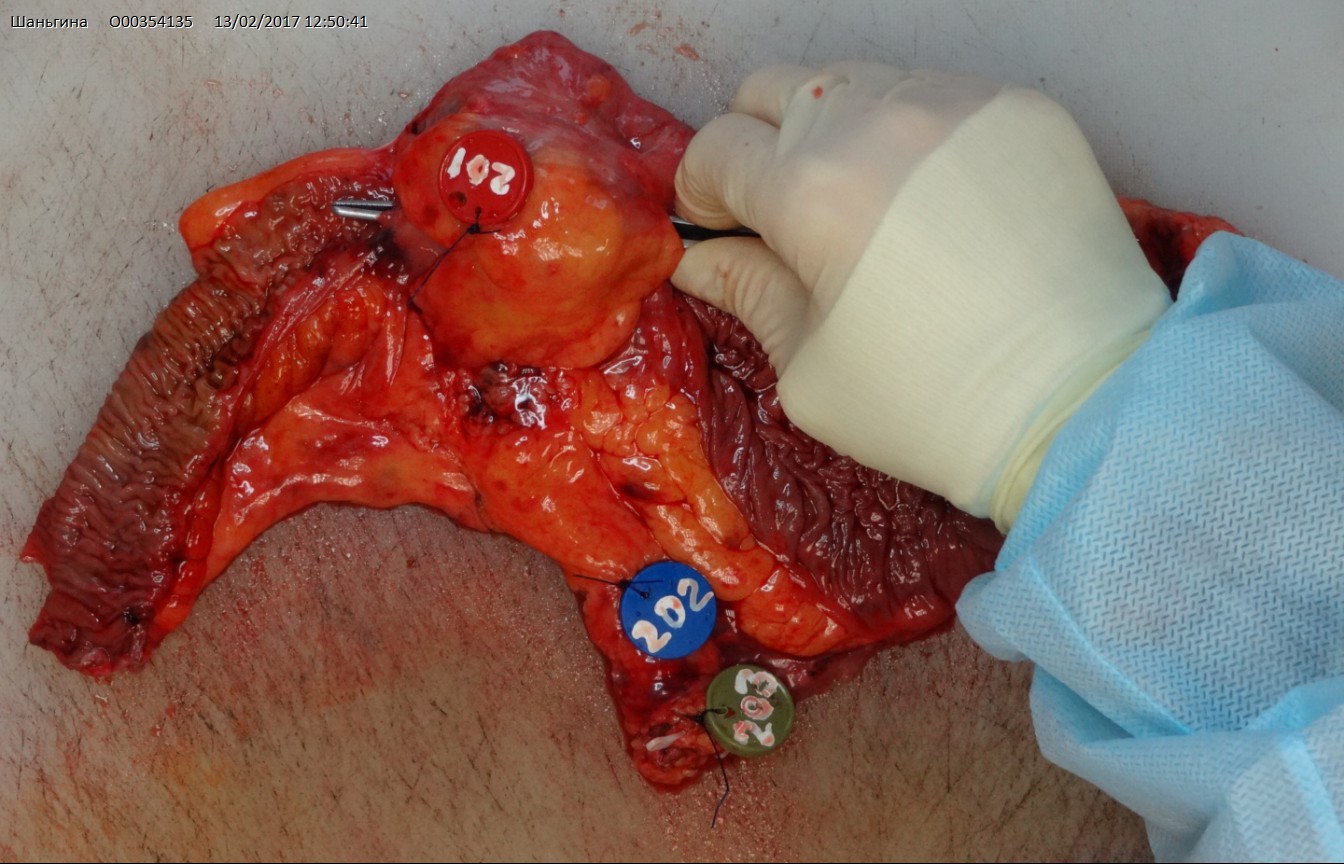


Fig. 5 Introduction of the swab to facilitate formalin delivery to the tumour


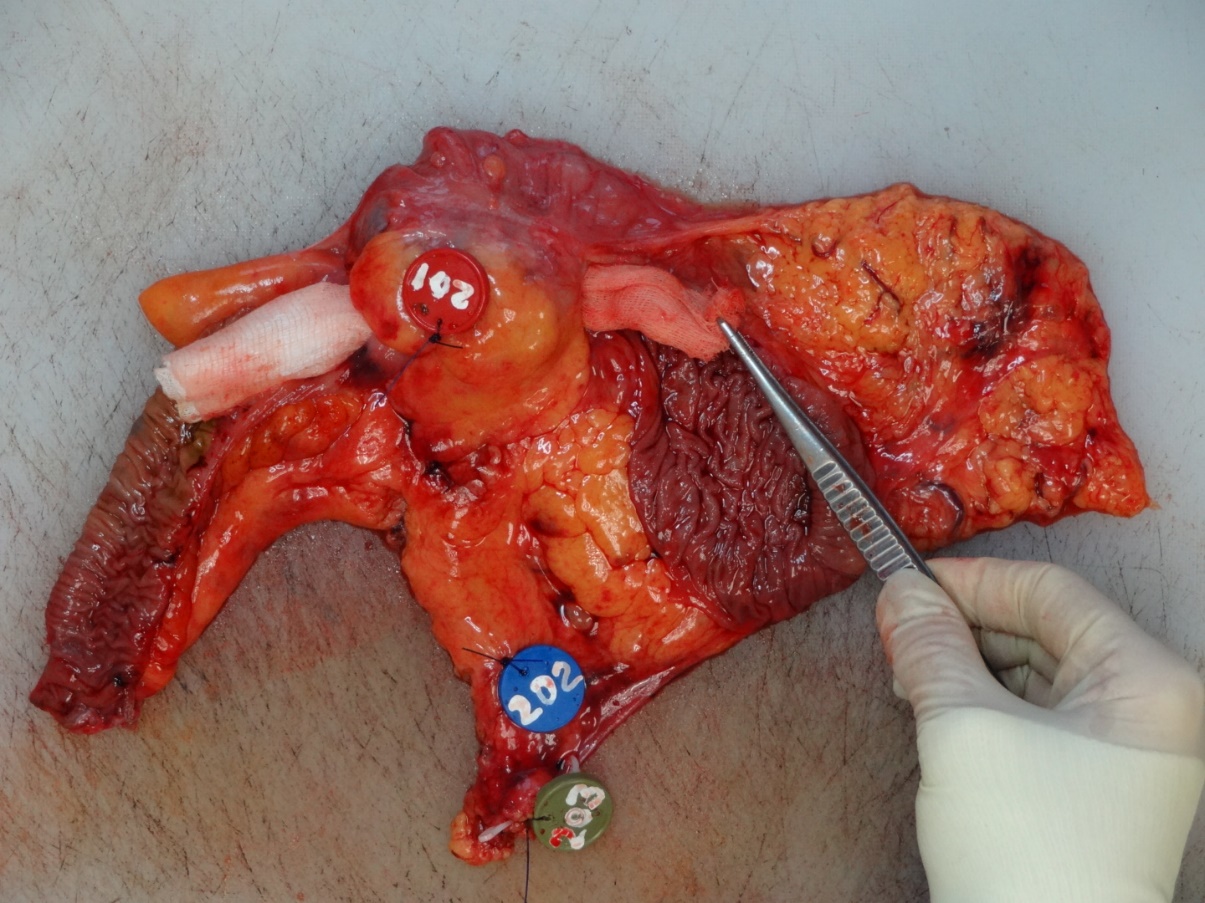


Fig. 6 Swab is located in unopened part of the lumen

If the tumour is located in the projection of mesocolic fascia, the latter is stained with a dye for further evaluation of circumferential resection margin during microscopic assessment. If the resected part of colon lacks mesocolic fascia or the tumour is located not in the projection of the fascia, distance from the tumour to serosa is measured in absence of direct tumour invasion to serosa (in mm or microns) (*Fig. 7*).

After the dye is dry, the specimen is immersed into 10% formalin buffered in 1:20 ratio for 12-24 hours.


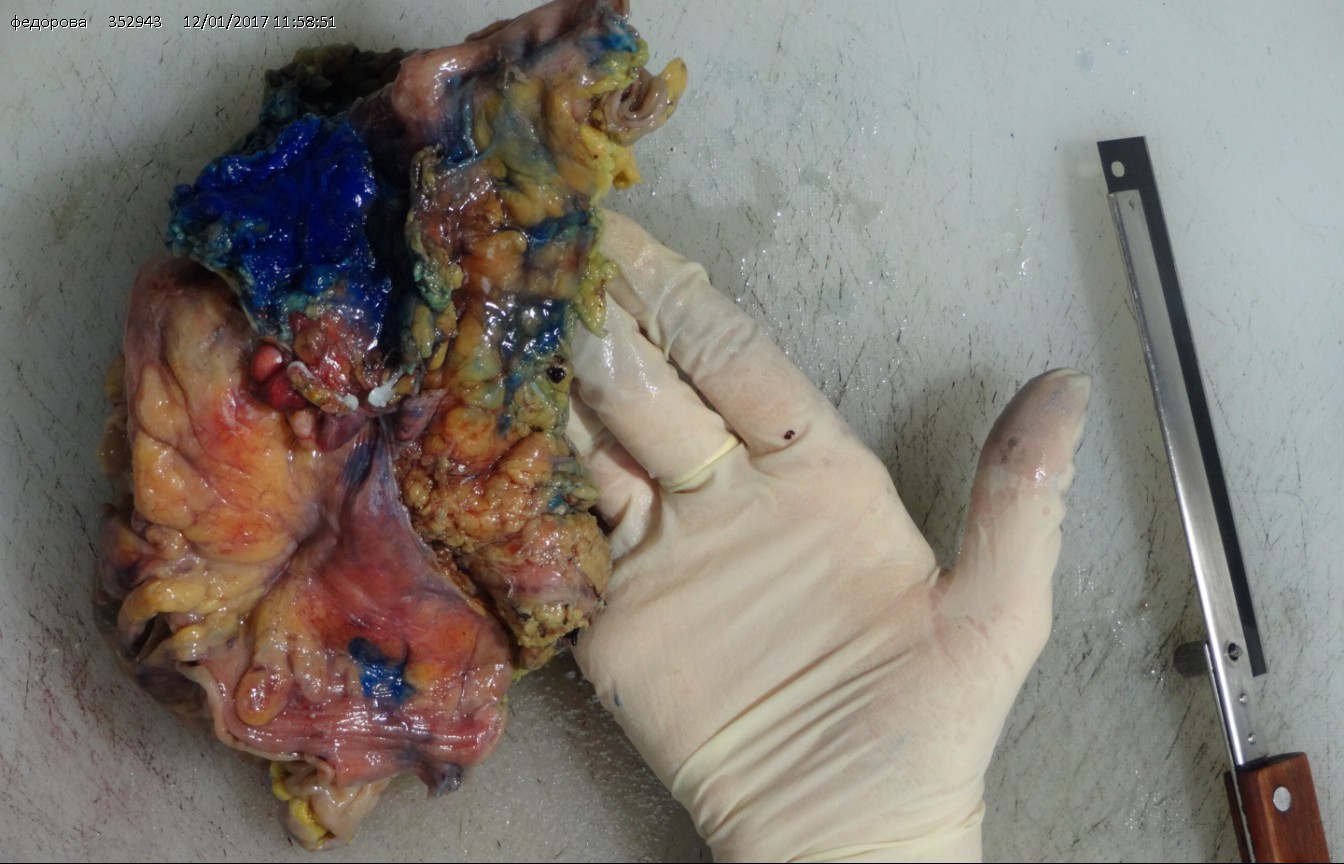


Fig. 7 Circumferential resection margin staining

2. Sectioning

After fixation the specimen is rinsed with sufficient amount of water, the swab is removed. If invasion of serosa is not suspected the lumen is opened completely with the cut in longitudinal direction. In case of serosa involvement suspicion 3 mm cuts are performed in perpendicular to bowel direction.

Sufficient samples including tumour with the minimal distance to circumferential margin or serosa are obtained. If the tumour with the margin does not fit to a cassette it should be evaluated in parts, relevant note is done in cassette description.

Lymph nodes are assessed according to group tags attached to the mesentery. Even in case of absence of macroscopically detectable nodes sufficient sample of the mesentery in tagged region is to be collected as lymph nodes may be revealed during microscopic evaluation. All lymph nodes of all groups are to be evaluated.

Example of macroscopic description:

16 cm long large bowel (caecum and ascending colon) with 10 cm of ileum, mesentery (area of the mesentery 7752 sq.mm), part of larger omentum 18x9x1 cm. CME of good quality. Distance from a. ileocolica ligation level to bowel wall – 10 cm, to tumour – 13 cm. Proximal distance is 16 cm, distal – 13 cm. Tumour is semi-circular, 8 cm in the largest measurement, 6 cm along the bowel. Invades paracolic fat.

Microscopic assessment:

- Histological type of tumour (WHO 2010 classification) and differentiation

- Depth of tumour invasion

- Extent of tumour lymphoid infiltration

- Presence of intra and/or extramural peri- and/or intraneural invasion

- Presence of intra and/or extramural lymphovascular invasion

- Tumour budding grade (Bd1, Bd2, Bd3 – according to consensus conference, Bern, 2016)

- Minimal distance to mesocolic fascia or serosa if tumour is located not in the projection of mesocolic fascia

- Status of margins

- Lymph nodes assessment in groups: presence/absence of metastases (number of metastatic lymph nodes and number of studied nodes for each group. If applicable – distance from lymph node metastases to circular resection margin is noted

- ICD-O code, pTNM stage.

Example of microscopic description:

Moderately differentiated adenocarcinoma invading paracolic fat with significant intratumoural infiltration, extramural peri- and intraneural invasion. No lymphovascular invasion. Tumour budding – 2. Minimal distance from tumour to mesocolic fascia 2.7 mm. Margins are free from tumour cells. Metastasis in 1 out of 10 lymph nodes of 201 group, 2 lymph nodes of 202 group and 6 lymph nodes of 203 group free of metastases. Large omentum of normal structure.

ICD-O code 8140/3, pT3N1a

# Appendix S3 Visits schedule

|  | Screening | Randomization | Surgery | Postoperative period^j^ | 30 days | 3 months | 6 months | 12 months | 2 years | 3 years | 4 years | 5 years |
| --- | --- | --- | --- | --- | --- | --- | --- | --- | --- | --- | --- | --- |
| Informed consent | √ |  |  |  |  |  |  |  |  |  |  |  |
| Examination/Patient history | √^a^ |  |  |  |  |  |  |  |  |  |  |  |
| Examination/phone call |  |  |  |  | √^k^ | √* | √* | √* | √* | √* | √* | √* |
| Colonoscopy | √^b^ |  |  |  |  |  | √ | √ | √ | √ | √ | √ |
| Histological confirmation | √^c^ |  |  |  |  |  |  |  |  |  |  |  |
| CT chest and abdomen | √^d^ |  |  |  |  |  | √ | √ | √ | √ | √ | √ |
| CEA | √^e^ |  |  |  |  | √* | √* | √* | √* | √* | √* | √* |
| Laboratory investigations | √^f^ |  |  |  |  |  |  |  |  |  |  |  |
| Anaesthetist/medical consult | √^g^ |  |  |  |  |  |  |  |  |  |  |  |
| Quality of life assessment | √^h^ |  |  |  | √ | √ |  | √ |  | √ |  | √ |
| Surgery planning | √^i^ |  |  |  |  |  |  |  |  |  |  |  |
| Adverse events |  |  | √ | √ | √ | √ | √ | √ | √ | √ | √ | √ |

* - phone call, examination and CEA blood test are performed every 3 month during follow-up period

**a** - during examination demographic data is collected including patient's contact number as well as of next of kin and one more relative or friend to facilitate further follow-up. Examination includes height and weight measurements, haemodynamic parameters (pulse and blood pressure), respiratory rate are assessed, general state of a patient (if patient is unwell - the cause is noted), examination of organ systems is performed (skin, ENT, thorax, breasts, abdomen, genital organs, musculoskeletal, vascular, neurological systems are assessed, lymph nodes are examined, auscultation of lungs and heart and rectal examination are performed). Full diagnosis is documented, if distant metastases are present - plan of treatment is documented. Data on comorbidities, previous surgeries and treatment is documented.

**b** - brief colonoscopy report is noted, including tumour location and if applicable T stage, judging by colonoscopy only. Investigations should not be earlier than 45 days prior to screening. If radiological investigations of colon were performed (CT-colonography or irrigoscopy) reports of these investigations are registered, noting location of the tumour according to radiology.

**c** - histological report should clearly indicate cancer. If morphological report is inconclusive another biopsy is necessary for patient inclusion.

**d** - CT chest and abdomen is performed for cancer staging. Distant metastases are detected, noting localisation and description of metastatic spread. In case of presence of metastases their resectability is determined. Lymph node metastases are detected in locoregional lymph nodes according to Japanese classification (N-staging). If the tumour is detected on CT size, length, T stage judging by CT and tumour localization is documented. Investigations should not be earlier than 45 days prior to screening.

**e** - CEA level is evaluated prior to treatment. Investigations should not be earlier than 45 days prior to screening.

**f** - laboratory investigations include, but are not limited to full blood count, biochemistry, urinalysis - additional test are ordered by attending doctor according to clinical circumstances and are used to assess patients health and if a patient is fit for surgery. In case of deviation of laboratory test results beyond referent intervals deviation is documented in CRF and comments are made on reason of deviation. For menstruating women or women with menopause less than 2 years pregnancy test is necessary - human chorionic gonadotropin level test in urine or blood.

**g** - anaesthetist or medic assess contraindications for surgery, evaluate patient on ECOG and ASA scales.

**h** - quality of life is assessed via questionnaires QLQ-C30 (general health) and QLQ-C29 (colorectal cancer module) filled in by patients.

**i** - registered in the study surgeon is assigned to perform surgery. Planned approach is noted - laparotomy, laparoscopy, etc. Based on preoperative data planned procedure is documented (right hemicolectomy, extended right hemicolectomy, left hemicolectomy, extended left hemicolectomy, sigmoid resection). Vessels, which are to be ligated according to tumour location, are documented. These vessels are to be dissected in D2 or D3 area depending on randomization group. Randomization group does not interfere with colonic resection extent, but only determines the area of ligation of vessels mentioned before randomization. Prior to randomization plan on distant metastases resection should be formulated.

**j** - postoperatively main parameters of patients' recovery are registered. Dates of admission and discharge, date of transfer to a ward from the ICU, date of independent standing and walking, date of first peristalsis, wind and stool, date of oral food and fluids intake are registered. Postoperative complications are documented and described according to Clavien-Dindo classification.

**k** - after 30 days complications of surgery or treatment are documents. If complications are present they are staged according to Clavien-Dindo classification. If staging investigations were performed - their results are noted.
